# Diffraction-geometry refinement in the *DIALS* framework

**DOI:** 10.1107/S2059798316002187

**Published:** 2016-03-30

**Authors:** David G. Waterman, Graeme Winter, Richard J. Gildea, James M. Parkhurst, Aaron S. Brewster, Nicholas K. Sauter, Gwyndaf Evans

**Affiliations:** a STFC Rutherford Appleton Laboratory, Didcot OX11 0QX, England; b CCP4, Research Complex at Harwell, Rutherford Appleton Laboratory, Didcot OX11 0FA, England; c Diamond Light Source Ltd, Harwell Science and Innovation Campus, Didcot OX11 0DE, England; d MRC Laboratory of Molecular Biology, Francis Crick Avenue, Cambridge CB2 0QH, England; e Lawrence Berkeley National Laboratory, Berkeley, CA 94720, USA

**Keywords:** global refinement, *DIALS* framework, centroid refinement

## Abstract

Rapid data collection and modern computing resources provide the opportunity to revisit the task of optimizing the model of diffraction geometry prior to integration. A comprehensive description is given of new software that builds upon established methods by performing a single global refinement procedure, utilizing a smoothly varying model of the crystal lattice where appropriate. This global refinement technique extends to multiple data sets, providing useful constraints to handle the problem of correlated parameters, particularly for small wedges of data. Examples of advanced uses of the software are given and the design is explained in detail, with particular emphasis on the flexibility and extensibility it entails.

## Abstract 2

A comprehensive description of the methods used within the *DIALS* framework for diffraction-geometry refinement using predicted reflection centroids is given. Examples of the advanced features of the software are provided.

## 1. Introduction   

The successful integration of single-crystal diffraction data depends on the accurate prediction of Bragg spot locations on area-detector images. An initial model for the diffraction geometry may be constructed from metadata provided with the diffraction images (Parkhurst *et al.*, 2014 ▸) or provided by the user. This starting model is completed by estimating crystal parameters, which are usually derived from data by an autoindexing procedure, such as that of Steller *et al.* (1997 ▸). This model is rarely sufficient for accurate prediction throughout a data set. Thus, a crucial step in data processing is to refine the geometrical model by procedures that minimize the discrepancies between spot locations observed on the image data and their locations as predicted from the model.

The refinement procedures employed by current software differ in their details, yet share much common ground. In contrast to macromolecular structure refinement, the task is often highly overdetermined, with a large number of observations (residuals) compared with the relatively small set of model parameters. Typically, these residuals consist of distances in a two-dimensional space linked to the area-detector surface (Leslie, 2006 ▸). For a rotation series, a third dimension expressed in terms of rotation angle, or depth within the series of diffraction images viewed as a stack, is also commonly used (Kabsch, 2010*b* ▸; Pflugrath, 1997 ▸). Additional terms such as reciprocal-space distance between the predicted and apparent scattering vector may also be included (Paciorek *et al.*, 1999 ▸). Most packages form a least-squares target function from these residuals and perform optimization using standard methods of nonlinear least-squares minimization. This type of refinement, in which the target function is expressed in terms of the discrepancies between predicted and observed spot locations, is conventionally called positional or centroid refinement.

A separate type of refinement may also be performed, which can improve the accuracy of unit-cell parameter estimates independently from detector positional parameters. This type of refinement uses as its target the degree of partiality of reflections recorded on two or more sequential images. The measure of spot partiality requires both a model for the rocking curve of the reflection and the integration of the complete intensity of that spot (Winkler *et al.*, 1979 ▸; Rossmann *et al.*, 1979 ▸; Leslie, 2006 ▸). As such, it can only be performed after integration has taken place, and is thus known as post-refinement. Positional and partiality refinement tasks may be treated separately (Leslie, 2006 ▸; Kabsch, 2010*b* ▸; Messer­schmidt & Pflugrath, 1987 ▸) or together in a joint refinement (Otwinowski *et al.*, 2012 ▸). The refinement of all parameters simultaneously with a sufficiently information-rich target function is known to be effective (Pflugrath, 1997 ▸; Kabsch, 2010*b* ▸; Paciorek *et al.*, 1999 ▸). However, subsets of the full parameter set may also be refined separately. For example, *MOSFLM* refines detector parameters using only a positional residual and uses a separate unit-cell refinement step to improve the crystal parameters by post-refinement. This ensures that the accuracy of refined unit-cell parameters is not affected by correlations with parameters of the beam and detector models.

In this paper, we discuss the implementation of three-dimensional centroid refinement algorithms within the *DIALS* framework (Waterman *et al.*, 2013 ▸). Our software provides global parameter refinement across one or more data sets, using a generalized model for diffraction geometry that can be applied to a wide variety of real instruments. Many of the core algorithms closely follow published methods and guidelines based on decades of expertise in the field, in particular proposals arising from the EEC Cooperative Programming Workshop on Position-Sensitive Detector Software (Bricogne, 1986*a* ▸,*b* ▸, 1987 ▸). The description of *DIALS* refinement here will necessarily repeat elements of that scheme, but our focus is on features unique to our software, including multiiple data set refinement, smoothly varying crystal parameters, extensible parameterization including the handling of multi-panel detectors, and an object-oriented design that facilitates the exchange of components such as the minimization engine. The described software is packaged together into a command-line program within the *DIALS* framework, called *dials.refine*.

## 2. Centroid refinement   

A central tenet of the *DIALS* framework is modularity, such that algorithms may be exchanged to alter or extend the capability of the software. A key issue this raises is the scope of each task. For instance, the geometry refinement should not include models that naturally fall within the scope of integration and hence become dependent on them. Those aspects of the global model that affect spot size and shape, such as the crystal mosaicity and beam divergence, are more appropriately dealt with by the part of the software that either learns or otherwise constructs models of the reflection profiles. This is a useful distinction, because for rotation-scan data these parameters do not alter the central impacts determining the recorded reflection position, only the general impacts that determine the reflection extent (Duisenberg *et al.*, 2003 ▸). This distinction does not hold for data sets consisting of still shots, because in general the reflecting condition for a central impact is not met on a still. In this case, the distribution of observed spots, and their partiality, is inescapably a function of general impact parameters. In this work, we restrict our description of geometry refinement to rotation experiments, and thereby to refinement of parameters that affect the reflection centroids, whilst making no assumption about parameters that contribute to spot size and shape.

This distinction excludes the traditional post-refinement residual within the global target function, as this necessarily includes a spot-profile model. Nevertheless, we find that refinement based on centroids alone is sufficient for accurate reflection prediction in integration, particularly when data sets are fine-sliced in comparison with the reflection rocking curve, yielding more accurate empirical centroid estimates. Furthermore, the separation of centroid refinement from profile refinement allows the use of *DIALS* refinement alongside any integration algorithm. *DIALS* is first and foremost a framework rather than a monolithic integration program (Waterman *et al.*, 2013 ▸) and therefore facilitates the construction of various alternative workflows for performing data-reduction tasks. Thus, implementation of traditional post-refinement algorithms within the *DIALS* framework remains an option for future work.

## 3. Reflection prediction with general diffraction geometry   

**Figure 1:**
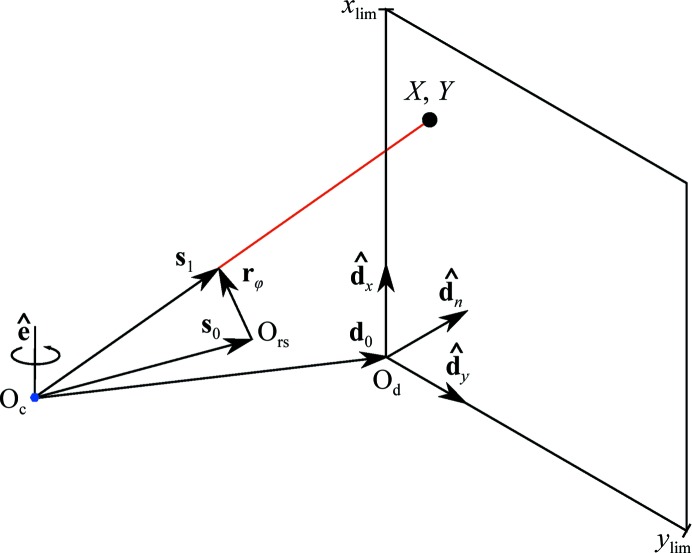
The diffraction geometry of the simple central impact model used by *DIALS*. Vectors are expressed in either a reciprocal-space or real-space laboratory frame. The rotation axis 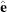 intersects the origin of laboratory space within the crystal at O_c_ and defines a right-handed rotation. The direct-beam wavevector **s**_0_ passes through O_c_ and defines the origin of reciprocal space O_rs_ at its tip. A reciprocal-lattice point rotated by an angle φ to touch the surface of the Ewald sphere is shown by **r**_φ_. The diffracted beam wavevector **s**_1_ extends from O_c_ to the tip of **r**_φ_. Further projection of this vector leads to an intersection on a detector plane at the position marked (*X*, *Y*). The detector plane is described by three vectors: **d**_0_, which defines the origin of the detector coordinate system O_d_ at one corner of the plane, and a pair of mutually orthogonal unit vectors 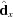 and 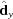 defining a Cartesian plane. The detector normal vector 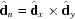 completes the detector coordinate system basis. The position (*X*, *Y*) is recorded if it is inside the pair of limits (*x*_lim_, *y*_lim_).

The general diffraction geometry for central impacts is shown in Fig. 1 ▸. The use of arbitrary vectors to describe the experimental geometry avoids limitations caused by adherence to an idealized geometry or coordinate system for a particular experimental method. The description that follows is a useful abstraction for correctly capturing the geometries found in any experimental diffractometer. Also, the implementation details of particular pieces of hardware play no role in the description of diffraction in these general terms. This design dates back to the seminal workshops at which a device-independent version of the program *MADNES* was planned (Bricogne, 1986*a* ▸,*b* ▸, 1987 ▸), and directly influenced the *dxtbx* software used by *DIALS* (Parkhurst *et al.*, 2014 ▸). Here, we follow closely the reports associated with that workshop and repeat some of the relevant mathematical working here for clarity, as unfortunately these documents are not widely available.

As described previously (Parkhurst *et al.*, 2014 ▸), a single flat-panel detector may be modelled by a plane positioned over the sensitive surface like a thin film. This abstract description of a detector panel is free of any hardware-specific effects, such as parallax and distortion corrections, as these are encapsulated within a tailored pixel-to-millimetre mapping function for that detector. For our purposes, it suffices to predict the point of intersection of a scattered ray, **s**_1_, with this plane. This is conveniently expressed using a projection along the direction **s**_1_ by a scale constant α, to meet the plane defined by unit vectors 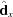 and 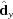, which are usually aligned with the ‘fast’ and ‘slow’ directions of the image array, respectively, for convenience. For detectors where these directions are not strictly orthogonal, another basis could be chosen, for example by aligning 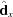 with the fast direction and 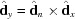, where 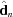 is normal to the detector plane. If **d**_0_ defines the origin of the detector coordinate system then the projection can be expressed as
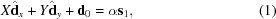
or, in matrix form, 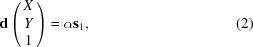
where 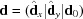 and therefore 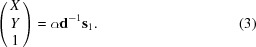
Defining the detector projection matrix 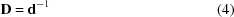
and a vector 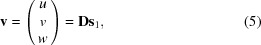
this can be rewritten as 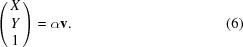
The point (*X*, *Y*) in millimetres on the detector plane is then represented by homogeneous coordinates (*u*, *v*, *w*) such that *X* = α*u*, *Y* = α*v* and *w* = 1/α. Therefore, 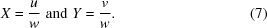

*X* and *Y* can then be obtained from calculation of **D** and knowledge of **s**_1_. The former comes from the orientation of the detector plane and, referring to Fig. 1 ▸, the latter is constructed as **s**_1_ = **s**_0_ + **r**_φ_.

The vector **r**_φ_ is related to the reciprocal-lattice vector in the initial orientation, **r**_0_, by 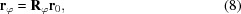
where **R**_φ_ represents a rotation about the axis 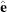 by an angle φ. Expanding this using Rodrigues’ rotation formula gives 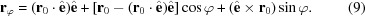
This expression is equivalent to that presented in §2.2 of Kabsch (2010*b* ▸). *DIALS* follows the *XDS* method to solve this for the φ angles of up to two intersections of **r**_φ_ with the Ewald sphere. To complete the description of reflection prediction, the reciprocal-lattice vector **r**_0_ is calculated for each integer index vector **h** using the standard relation
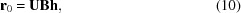
where **U**, the orientation matrix, is a rotation matrix and **B**, the reciprocal-space orthogonalization matrix, contains the components of the reciprocal-space unit-cell vectors in an orthogonal coordinate frame fixed to the crystal, such that **B** = (a*|b*|c*).

Following the above, spot positions can be predicted in detector space and rotation angle in the form of the triplet (*X*, *Y*, φ). By analysis of the diffraction images the equivalent information can be extracted for the observed spots, using a detector-specific pixel-to-millimetre function. In order to perform refinement, derivatives of the calculated (*X*, *Y*, φ) are needed with respect to the parameters of the model. Abstract expressions for these derivatives are determined that are not dependent on the actual parameterization chosen, so that alternate parameterizations can easily be applied. The calculation of these derivatives is described in detail in Appendix *A* [App appa].

## 4. Model parameterization   

The *DIALS* refinement module makes an explicit distinction between the experimental models (beam, crystal, goniometer and detector) and the parameterizations of these models. The models are used throughout the *DIALS* framework and are abstract descriptions of the physical components that they represent, intended to be generally applicable to a wide variety of experiments and to be used by all algorithms written within the *DIALS* framework. A model parameterization, in contrast, is relevant only within refinement. Each parameterization attaches to its model for the duration of the refinement procedure, providing a means to express the state of the model from the values of its parameters, to calculate first derivatives of this state and to update the model after each step of the refinement algorithm. This distinction enables great flexibility, as alternative parameterizations may be applied to a particular model to control the behaviour of refinement. This may be used to represent different levels of prior knowledge about an experiment. For example, the default detector parameterization in *DIALS* represents the position and orientation of each detector plane with six degrees of freedom. For some instruments it may be appropriate to restrict relative offsets of individual planes to translations, or to specify certain known mechanical axes about which operations of translation or rotation may take place. Such cases would be accommodated by providing an alternative parameterization to attach to the core detector model. This alternative parameterization would be guaranteed to affect only the behaviour of refinement in *DIALS*, because the core detector model remains unchanged.

Alternative parameterizations offer great flexibility, but it is recognized that the majority of cases are well served by the default set of model parameterizations provided by *DIALS*, the details of which are summarized in Table 1 ▸. The gonio­meter model is not currently parameterized in *DIALS*, as it is assumed that the rotation axis is known within the laboratory frame. Although the standard set consists of 18 parameters, it is not possible to refine them all simultaneously: one parameter must be fixed relative to the laboratory-frame coordinate system, otherwise it would be possible to rotate the whole experiment freely around the rotation axis. A second direction, off the rotation axis, must also be specified in the laboratory frame to disallow this. In fact, by convention we adopt the imgCIF coordinate system (Bernstein, 2005 ▸), in which the principal goniometer axis coincides with the 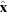 axis of a right-handed set and the 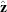 axis is defined such that the beam vector lies in the 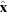
–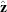 plane with 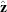 pointing towards the beam source rather than towards the detector. To adhere to this, in normal usage the parameter μ_1_ = 0 is fixed to allow beam movements only within this plane. This behaviour is merely conventional: it is possible for the user to fix any other parameter, and define the goniometer axis as some other arbitrary unit vector, in order to parameterize the experiment with respect to a different coordinate frame. It is required only that the origin of the coordinate system is formed at the point of intersection between the beam and the crystal, and that the system forms a right-handed orthonormal basis. In addition to prohibiting free rotation of the experiment, the beam wavelength (parameterized as the wavenumber, the length of the **s**_0_ vector) is usually fixed, as this is fully correlated with the unit-cell volume and is typically measured to better than one part in 1 × 10^5^ at most synchrotron beamlines. In situations where the cell is known accurately but the wavelength is unknown, this cell can be fixed and the wavelength allowed to refine, for example for beamline calibration with a well characterized small-molecule crystal.

Each model parameterization designates some aspect of its model as its ‘state’. As shown in Table 1 ▸, this is either a vector or a 3 × 3 matrix. At initialization, parameters are calculated such that the model parameterization reproduces the current model state and is able to compose future states as the parameter values change. The translational and rotational parameters are always associated with an axis along or about which their action takes place. To avoid dependence on the laboratory-frame definition, for the beam and detector parameterizations these axes are constructed from the initial geometry of the models. For example, the detector *p*_0_ parameter is a distance along a vector normal to the initial detector plane. Axes such as these are stored inside the model’s parameterization object and persist for the lifetime of the refinement procedure, so although the detector plane orientation may change during the course of refinement, the *p*_0_ parameter always acts along this initially determined direction. As a result, this scheme works equally well with any orientation of the laboratory-frame axes, ensuring that the algorithm is unaffected by this arbitrary choice. In this work, the stored axes that are determined from the initial geometry of the model are indicated using a superscript prime. One consequence of defining the action of parameters with reference to these axes is that absolute parameter values are not comparable between the output of individual refinement procedures if their initial geometries differ. This is not a deficiency, because the parameter values are not expected to carry any useful meaning outside of refinement. Rather, it is the set of refined models that form the output of a refinement procedure, and the states of these models that may be compared. An advantage of this approach is that the angle parameters describing the ‘misset’ orientation of the crystal from its initial or datum orientation can always be set equal to zero at initialization and be expected to remain small for any reasonable refinement task; therefore, the theoretical issue of gimbal lock is not met in practice.

In contrast to the beam and detector parameterizations, the axes of action for the crystal orientation parameterization are aligned with the laboratory-frame axes. The crystal orientation is determined by Euler angles in the Tait–Bryan convention (with rotation first around laboratory 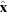, then 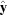 and finally 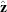). Any mutually orthogonal basis would suffice and the laboratory basis is chosen for simplicity. Unlike the other model parameterizations, the crystal unit-cell parameterization is not made in terms of lengths and angles of vectors, but instead using those elements of the reciprocal-lattice metrical matrix that are not constrained by space-group symmetry. This allows the use of code within the *Computational Crystallo­graphy Toolbox* (*cctbx*; Grosse-Kunstleve *et al.*, 2002 ▸) which automatically determines which parameters are free for each space group and setting, avoiding the use of special code to handle each case (Sauter *et al.*, 2006 ▸). Although elements of the reciprocal metrical matrix G* = 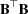 are used as parameters, the model state for the crystal unit cell is the reciprocal-space orthogonalization matrix **B** (Busing & Levy, 1967 ▸), using the convention defined previously (Gildea *et al.*, 2014 ▸). The derivatives of **B** with respect to the free elements of G* are calculated using *cctbx*. The derivatives of states of the other models (beam, crystal orientation and detector position and orientation) are detailed in Appendix *B* [App appb].

When composing new model states from parameter values, the order in which the parameter actions are applied is clearly important. For the beam model, a new state is composed using 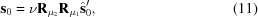
where the meanings of the parameters are as described in Table 1 ▸ and 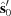 is the unit vector giving the beam direction in its initial orientation.

The crystal orientation is composed by forming a misset rotation matrix, 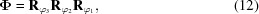
that operates on the datum orientation of the crystal **U**_0_, which is also a rotation matrix. These missets act in a coordinate frame fixed to the crystal (*i.e.* equivalent to the laboratory frame at goniometer rotation angle φ = 0) such that the crystal setting in the laboratory frame after a rotation by **R**_φ_ is given by 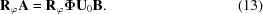



To parameterize a detector plane, a reference coordinate basis is formed from the orthonormal triplet 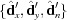, consisting of a pair of in-plane axes and the plane normal vector for the initial orientation, which may face either towards or away from the sample. The initial orientation angle parameters τ_1_, τ_2_ and τ_3_ are set to zero. A new orientation is composed from updated parameter values using the combined rotation 
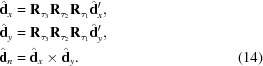



**Figure 2:**
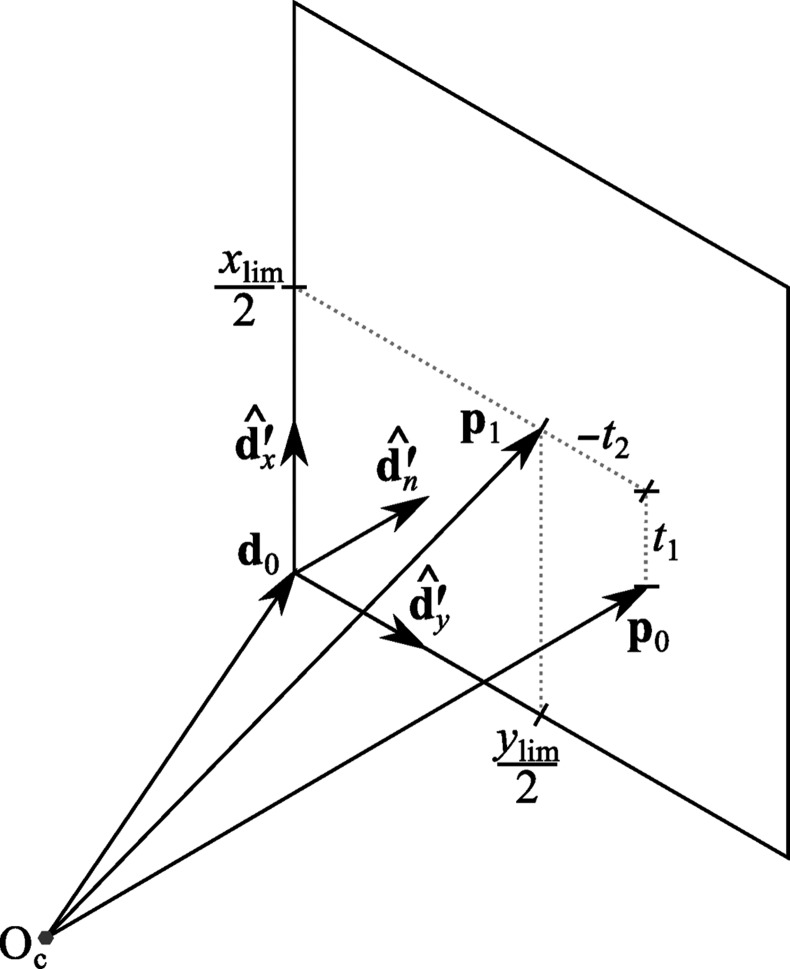
The definition of the detector-distance parameter requires a vector **p**_0_ from the origin of the laboratory frame within the crystal, O_c_, to a point on the detector surface along the initial detector normal vector 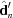. The length of this vector *p*_0_ defines the detector distance. The orthogonal shift parameters *t*_1_ and *t*_2_ are determined with reference to the point **p**_1_ at the centre of the detector plane, as set by the limits *x*_lim_ and *y*_lim_. Note that here *t*_2_ is negative as the shift required along 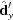 goes against the direction of this vector. Following location of the centre of the detector plane in its initial orientation, rotations determined by the orientational parameters are applied about the point **p**_0_. The action of these orientation parameters is not shown here.

The effect of the positional parameters can be shown by defining a pair of vectors marking points on the detector surface, as shown in Fig. 2 ▸. A vector normal to the initial detector plane, 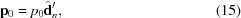
defines the detector ‘distance’ parameter *p*_0_, while a second vector includes the orthogonal translation parameters *t*_1_ and *t*_2_, 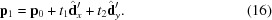
The vector **p**_1_ marks an arbitrary point on the detector plane in its initial orientation depending on the size of these shift parameters. To set the initial values of these parameters, a reference point on the detector surface must be chosen. By convention, this is set to be the centre of the detector as defined by its rectangular extent in the 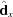 and 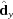 directions, 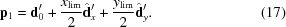
Comparison with (16) gives 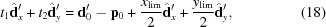
from which the translational shift parameters are derived, 
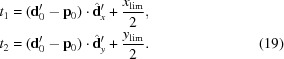



The vector 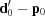 gives the fixed offset of the detector origin 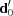 from the reference point 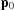. This vector is referred to as 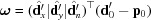 when expressed in the initial detector coordinate frame and will be reused to determine the updated detector origin below.

Having defined the translational parameters, rotations are then applied to **p**_1_ about the point **p**_0_. This point was chosen as the origin of rotation rather than the laboratory-frame origin to reduce correlation between the translational and rotational parameters. Firstly, **p**_1_ is rotated by an angle τ_1_ around the axis 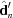. This axis passes through the laboratory-frame origin, so rotating either around point **p**_0_ or the origin is equivalent and simply gives 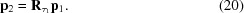
This is followed by a rotation of τ_2_ around the axis 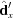, taken through the point **p**_0_,
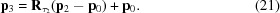
Finally, rotate by τ_3_ around the axis 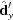 through point **p**_0_ and expand the terms: 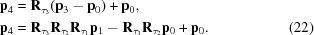



The updated reference point **p**_4_ together with the updated basis 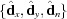 are used to find the new detector origin **d**_0_ from 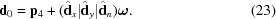
The vectors 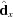, 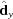 and **d**_0_ define the new detector matrix **d**.

It is trivial to extend this detector parameterization for a single detector panel to some group of panels which are not necessarily coplanar. The *dxtbx* library provides an extension of the simple detector model to represent a hierarchical detector in which panels are arranged into groups and form a tree structure (Parkhurst *et al.*, 2014 ▸). To parameterize a hierarchical detector, a level in the hierarchy must first be selected to specify which panel groups are to be moved as rigid units. Each panel group *k* specifies its own group frame with detector matrix **d** ^*k*^. The reference point for the group **p**_1_^*k*^ is chosen to be a point that intersects the group frame close to the centre of the group of panels. The composition of a new position and orientation of the group frame proceeds exactly as outlined above. When the group is updated with a new matrix **d** ^*k*^ the laboratory-frame positions and orientations of the panel children are updated automatically by the hierarchical model.

In addition to providing the means to compose new states for their parameterized models, the model-parameterization objects calculate the derivatives of these model states with respect to the chosen parameterization. These derivatives are passed to a higher level object that parameterizes the prediction equation, and they are combined as described in Appendix *A* [App appa]. Expressions for the derivatives of each model state with respect to the default set of parameters supplied within *DIALS* are presented in Appendix *B* [App appb].

## 5. Minimization   

Minimization requires three entities: a model function, a target function and a minimization algorithm. The model function for centroid refinement is the vector-valued reflection-prediction equation, which for some integer index vector **h** calculates the reflection centroid 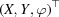 according to the model states **s**_0_, **U**, **B** and **d**, and a Boolean flag *e* ∈ {true, false} that determines whether the passage of the reciprocal-lattice point is entering or exiting the Ewald sphere,
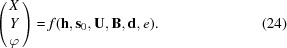



The first derivative of the predicted rotation angle φ with respect to any parameter becomes undefined when the triple product 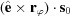 approaches zero (see equation 40 in Appendix *A* [App appa]). A geometrical interpretation of this term is the volume of the parallelepiped formed by the rotation axis 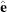, the reciprocal-lattice vector **r**_φ_ and the beam vector **s**_0_, which approaches zero for reflections that are close to the rotation axis. These reflections are also expected to have poorly determined φ centroids as the Lorentz factor exhibits the same asymptotic behaviour. To avoid the inclusion of reflections that destabilize the φ derivatives this volume is calculated, and any reflection with a volume below a user-defined cutoff (with a default value of 0.05) is discarded.

The target function consists of the weighted sum of squared residuals between the calculated and observed centroid positions (*X*, *Y*, φ) over the set of *n* reflections used in refinement: 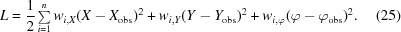



The weighting scheme may be selected by the user from a number of strategies, of which two are currently provided for the refinement of rotation scans. The simplest is a constant weighting scheme in which *w*_*i*,*X*_, *w*_*i*,*Y*_ and *w*_*i*,φ_ may be set to user-defined constant values, *w_X_*, *w_X_* and *w*_φ_. This may be useful when estimates of centroid variances are not available (such as when taking as input centroids determined by external software). However, given the centroid positions and their variances, as determined by the program *dials.find_spots*, the default behaviour in *DIALS* is a statistical weighting scheme whereby the *w_i_* terms are set equal to the inverse variance estimates of the observed centroids. Assuming that these are accurately determined, each term then contributes a unitless quantity to the target function.

The task of finding parameters that minimize *L* is directly amenable to methods of nonlinear least squares. This requires the first derivatives with respect to each parameter, *p*, given by
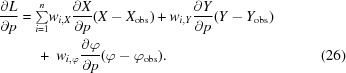
These methods are applied using code already available in *cctbx* within the *lstbx* subpackage (Bourhis *et al.*, 2015 ▸). A simple Gauss–Newton algorithm provides fast rates of convergence; however, this is subject to failure when the effects of pairs of parameters on the model are highly correlated (Reeke, 1984 ▸). Alternatively, the Levenberg–Marquardt algorithm may be used, improving the stability at the cost of a few more steps to convergence. This algorithm is chosen as the default for *DIALS* because of its robustness. Other methods to minimize the target function may also be employed, as long as they require only its first derivatives and can be adapted to adhere to a particular interface specified by the refinement engine base class. Alongside the nonlinear least-squares algorithms, the opportunity to use the limited-memory Broyden–Fletcher–Goldfarb–Shanno (L-BFGS) method (Liu & Nocedal, 1989 ▸) is provided. This is of particular value for problems involving a large number of parameters, where constructing the normal matrix is inefficient (discussed further in §[Sec sec8]8).

By contrast, the usual problem of refinement for a single rotation scan of a protein crystal is typically highly overdetermined, with a small number of parameters (tens to hundreds) and a large number of observations (many thousands). In such cases it can be appropriate to take a random sample of the input data, if requested, rather than using all of the strong reflections in a data set, to produce equivalent results in a shorter execution time. For example, a test of scan-varying refinement using the data publically available at http://dx.doi.org/10.5281/zenodo.10271 (Winter & Hall, 2014 ▸), using all 101 841 reflections accepted for refinement and a total of 22 parameters, completed in 26 s on a Linux desktop with an Intel Core i7 processor operating at a 3.07 GHz clock speed, using a single process. The refined model produced r.m.s.d.s of 0.26 pixels in *X*, 0.22 pixels in *Y* and 0.13 images in φ. Using 50 reflections per degree of the scan (4049 in total) allowed refinement to complete in 7 s with the same r.m.s.d. values to two decimal places.

## 6. Outlier rejection   

The least-squares method is neither robust nor resistant, which means that the refined parameter estimates can be highly sensitive to extreme values in the data. These values may result either from long-tailed error distributions or the presence of outliers in the data (Prince & Collins, 2006 ▸). The squaring of residuals in the target function (25) magnifies the effect of extreme data points, such that the inclusion of even a single severe outlier can dominate the minimization procedure. As it is the form of the target function that holds this property, altering the choice of minimization algorithm cannot improve robustness or resistance.

Methods of robust estimation have been formulated, including the use of M-estimates (Huber, 1973 ▸), and this continues to be an area of active research in the field of robust statistics. However, all such methods introduce additional algorithmic complexity and suffer other disadvantages such as invalidating the procedure that will be presented in §[Sec sec10]10 for calculating parameter error estimates. A simpler approach, which is appropriate when dealing with approximately normally distributed data contaminated by outliers, is to identify and remove outliers first and then proceed with the usual minimization of the target function calculated over the accepted subset of reflections. If only genuine outliers are removed, the resulting parameter estimates and their errors do not accrue bias by this procedure. In practice, it can be difficult to distinguish outliers, especially if the true distribution of errors is not known *a priori*. Nevertheless, the inclusion of outliers can be so disastrous that in their presence imperfect outlier rejection may be better than no outlier rejection at all.

In accordance with the spirit of flexibility adopted by *DIALS*, alternative outlier-rejection algorithms are offered, with parameters available to the user to control their behaviour. The simplest of these algorithms is inspired by the box plot, a nonparametric display of the variation of univariate data. In this method, the median and the interquartile range of the data are first calculated, giving robust estimates of both location and scatter. Outliers are classified as any point lying outside some multiple of the interquartile range beyond the first and third quartiles. When this multiple is equal to 1.5 (the default) these boundaries are equal to Tukey’s inner fences (Tukey, 1977 ▸). For rotation data this analysis is performed on each of the residuals in *X*, *Y* and φ. Reflections are marked as outliers if any of the associated residuals falls outside the boundaries. Taken in combination, these boundaries therefore form a cuboid in the space of the residuals. This method is very fast but has one major drawback, which is that it ignores the multivariate nature of the centroid residuals.

For a multivariate data set, one method to determine probable outliers is to calculate the Mahalanobis distance (Mahalanobis, 1936 ▸) of each of the points, with a choice of distance cutoff marking the boundary between accepted and outlier data. The Mahalanobis distance is a multivariate generalization of the number of standard deviations that a point lies away from the mean of a distribution. If the data are normally distributed then the squared Mahalanobis distances follow a χ^2^ distribution, so an appropriate boundary distance can be taken from the quantile function of the χ^2^ distribution with the appropriate number of degrees of freedom (three for rotation data). By default the 97.5% level is used to determine the distance cutoff, but other levels may be selected by the user. This method takes into account correlations in the data set and, in contrast to the previous method, the boundary forms a smooth ellipsoid in the space of the residuals. By itself, the Mahalanobis distance calculation is not resistant to the presence of outliers in the data; therefore, it is essential to use a robust method to estimate the central tendency and covariance of the data set. We use an implementation of the *FAST-MCD* algorithm to calculate the minimum covariance determinant estimator of these quantities (Rousseeuw & Driessen, 1999 ▸) with corrections applied to the covariance matrix (Croux & Haesbroeck, 1999 ▸; Pison *et al.*, 2002 ▸).

The third outlier-rejection algorithm available in *DIALS* refinement is described in Sauter & Poon (2010 ▸). This method uses only the (*X*, *Y*) residuals on the surface of the detector and is of particular interest for still-shot or narrow-wedge data collections where the φ residual is poorly determined.

## 7. Global refinement   

A common mode of operation in current software, as exemplified by the programs *MOSFLM* (Leslie & Powell, 2007 ▸) and *XDS* (Kabsch, 2010*a* ▸), is to index and refine an initial model and then process a data set in a linear fashion from beginning to end of the sweep, alternating between refinement and integration tasks. The integration model is localized in φ by refinement tasks that are specific for a small wedge of data. The model may not be appropriate outside of this φ window owing to changes in the diffraction geometry such as the crystal setting angles. However, the refined model is adequate for spot prediction within this wedge of images and is taken as the starting point for refinement within the next φ window. This method may also ‘correct’ for deficiencies in the geometrical model that preclude a general representation for the full data set, such as enforced ideal geometry of the rotation method (with the spindle axis and detector plane both at right angles to the beam) where this is not strictly appropriate. This approach of alternating local refinement and integration was developed when typical data-collection experiments were slower than the computational processing, and analysis would start before data collection was complete. Processing only small wedges also ensured a reduced memory requirement, which was a key issue in the design of software for older hardware.

Modern rotation-method experiments carried out at third-generation synchrotron beamlines combine intense beams with fast-readout detectors, allowing shutterless data collection in which complete diffraction data may be collected in seconds (Broennimann *et al.*, 2006 ▸; Winter & McAuley, 2011 ▸). Characterization of a crystal and data-collection strategy calculation remain as important as ever, and online data analysis may allow data collection to be terminated early if diffraction quality fades (Zhang *et al.*, 2006 ▸). However, it is not generally realistic to expect high-quality data processing to be performed in tandem with, and at the same rate as, data collection. Given the rapid completion of most data collections, there is no significant overhead in finishing each collection before careful integration of these data. As we can expect there to be a complete data set present for analysis at the outset, this allows us to refactor the workflow of data-processing software. Rather than perform cycles of local refinement and integration during a linear pass through the data, we can consider the complete determination of a global centroid prediction model by a single refinement procedure prior to carrying out the integration.

**Figure 3:**
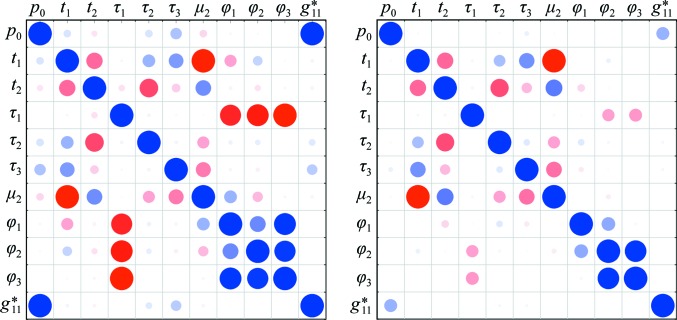
Both panels show a type of ‘corrgram’ (Friendly, 2002 ▸) providing a rapid visual indication of the sign and magnitude of correlations between columns of the Jacobian matrix used in the final step of nonlinear least-squares refinement. The Jacobian represents the sensitivity of the prediction formula to parameters of the model. Parameters are listed in order along the rows and columns of a grid corresponding to a square correlation matrix, with parameter names as defined in Table 1 ▸. The large blue circles seen down the leading diagonal indicate the perfect correlation between the effects of parameters with themselves, while large red circles show high anticorrelation. The colour and area of the circles between the extremes are related to the numerical value of the correlation coefficient, so that the most important effects stand out whereas low correlations fade to empty squares. The left panel was produced by *dials.refine* for refinement of a 1° data set recorded on a cubic polyhedrin crystal (Gildea *et al.*, 2014 ▸). The right panel results from multi-experiment joint refinement of five 1° sweeps of data consisting of a total of 16 indexed lattices with a shared beam and detector model. For clarity, only the crystal parameters of the same lattice as that treated alone on the left panel are shown. Comparison between the panels shows that the inclusion of multiple lattices significantly reduces the correlations between crystal parameters and other parameters of the model. For example, the correlation between the detector distance and the single-crystal lattice parameter is reduced from 0.995 to 0.323 by multiple lattice refinement.

Global refinement has some distinct advantages that stem from the application of prior knowledge of the experimental design. For example, we may know that the detector and beam did not move appreciably during the collection of a data set (or even multiple data sets). Rather than refining their parameters to different values per image, or periodically over the data-collection scan, we can use the global data to find the best-fitting parameter values as a whole. When wide wedges of data are available, global refinement also alleviates the problem of parameter correlation. Within a small φ range, the errors in certain parameters are highly correlated with others (see Fig. 3 ▸). This degeneracy makes it troublesome to obtain accurate parameter values, and may even lead to the failure of certain algorithms used to perform the minimization. For nonlinear least-squares methods, the latter may be avoided by applying eigenvalue filtering (Reeke, 1984 ▸; Bricogne, 1986*b* ▸) or singular value decomposition (Nocedal & Wright, 2006 ▸); however, this does not improve the accuracy of refined parameter values. Apart from the issue of parameter correlations, some parameters are poorly defined in a local region of the rotation scan, such as when a cell direction is close to parallel to the primary beam direction. In the case of a low-symmetry lattice it may not be possible to refine this unit-cell parameter unless data from another orientation are also available (Leslie, 2006 ▸). Within the local φ window, these issues may be of secondary concern, as the main role of the model parameters is simply to locate the measurement boxes correctly over the diffraction spots. Nevertheless, it is desirable to obtain accurate unit-cell parameters for downstream processing of the data, for which the power of global post-refinement is well recognized (Otwinowski & Minor, 1997 ▸).

Global refinement also enables potentially very fast integration procedures. Because the geometrical model for integration is determined prior to the integration procedure, this is readily separable and can make use of brute-force parallelism. This form of parallel execution is preferable to the batch execution of refinement and integration tasks with subsets of the full sweep, as in the latter case changes to the refined model may vary discontinuously from batch to batch and care must be taken to minimize the effect of this on the integrated intensities (Kabsch, 2010*a* ▸). Furthermore, the separation of refinement increases the flexibility of integration, as different algorithms may be compared under the same conditions.

## 8. Multiple-experiment refinement   

**Figure 4:**
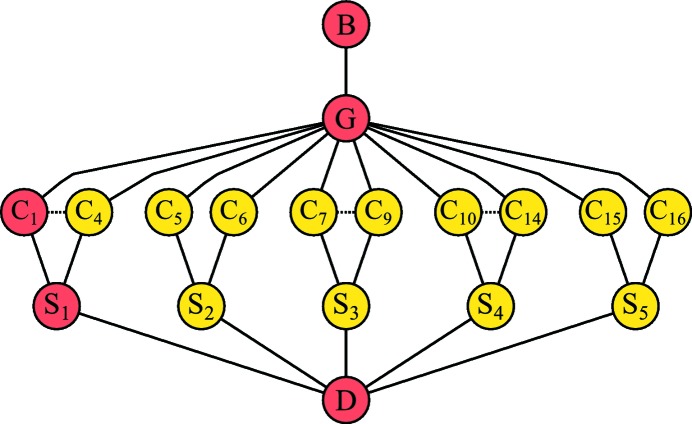
The global model used for a complex multi-experiment refinement procedure can be illustrated by a graph linking experimental models labelled as follows: B, beam; G, goniometer; C, crystal; S, scan; D, detector. A single experiment consists of a path from top to bottom connecting one of each type of model. Shown here are the jointly refined models for the polyhedra example discussed in the text. The experiment highlighted in red corresponds to the parameters displayed in Fig. 3 ▸. Where more than two crystals were present in a scan, the set of crystal models are shown as a range. All crystals within a range share the same connections, so for clarity only the extrema of the range are shown.

The approach of global refinement presented in §[Sec sec7]7 is applicable to data from throughout a single-crystal data set. For special cases, the idiom may be extended to a higher level to encompass data from multiple experiments in a single joint refinement. Here, the term ‘experiment’ has a precise meaning within the *DIALS* framework, and refers to a set of unique experimental models necessary to satisfy the diffraction condition and produce a consistent set of measured intensities. An experiment must therefore contain exactly one beam, one crystal and one detector model. It may also contain one goniometer and one scan model if the experiment is a rotation scan. It is possible to jointly refine multiple experiments if those experiments share one or more models. The commonest example is that of multi-lattice data, in which the experiments differ only in their crystal models. The use of *DIALS* multi-lattice refinement during the indexing of small wedges of multi-crystal data has previously been presented (Gildea *et al.*, 2014 ▸). Fig. 3 ▸ shows how the correlation between crystal and detector parameters for one of these small wedges is reduced when multiple lattices are refined together, and Fig. 4 ▸ illustrates the set of models used for joint refinement.

When the number of experiments in a refinement procedure is greater than one, some parameters included in the global model are specific only to particular experiments. This increases the sparseness of the problem, as these parameters contribute gradients of zero for all observations outside these experiments. For efficiency, sparse-matrix techniques may be used during the calculation of the normal matrix to avoid the explicit storage of these zero elements. For large numbers of experiments, calculating the normal matrix may be prohibitively inefficient. In those cases, use of the L-BFGS method is preferred. We are currently investigating the application of the *DIALS* refinement module to large problems, such as joint refinement of data sets consisting of multiple still shots, and will address these issues in more detail in the future.

## 9. Scan-varying crystal parameterization   

During data collection the crystal unit cell may change owing to radiation-induced structural changes. Changes in the illuminated crystal volume during rotation or translation may introduce undamaged regions into the beam, thereby impacting the effective unit-cell parameters. In addition, crystal movements or goniometer defects may lead to changes in the crystal orientation. Where these changes occur smoothly over the course of a rotation scan, the benefits of global refinement of parameters can still be obtained by explicitly including a smoothly varying parameterization of the crystal, expressed as a function of position in the contiguous series of images forming the scan. For this purpose a Gaussian smoother based upon code from *AIMLESS* (Evans & Murshudov, 2013 ▸) is used. This provides a simple and fast way to calculate smoothly changing values and gradients for an arbitrary parameter without assuming any particular functional form for the behaviour of that parameter. To set up the smoother, a number of equally spaced points are chosen throughout the scan, based on a user-configurable interval width.

Each of these points is associated with a Gaussian function and a scaling coefficient that becomes a refinable sub­parameter of the model. At any point along the scan the smoothed parameter value can be constructed by taking the Gaussian-weighted sum of the nearest three subparameter values. Initially the subparameters are all set to the same value, so that the smoothed parameter curve is flat over the whole scan. During refinement the subparameters are allowed to vary in order to reduce residuals. As each subparameter has a local effect in a Gaussian-weighted sense, this results in a smooth curve moving from one region of a scan to the next. This model is deemed appropriate to capture smoothly varying physical changes during data collection.

The degree of smoothing is controlled by the number of intervals into which the scan is divided. By default an interval width of 36° is used, as this was found to provide suitable results in practice for data sets collected at Diamond Light Source. The number of sampling points is chosen to be an integer such that the spacing between sample points is close to the specified value, with the extreme points lying outside of the scan range. Other parameters of the smoother are derived automatically. For example, the number of nearest points over which to average is set to three, as no significant advantage was observed by increasing the number of points. In addition, the width of the Gaussian functions is linked to the interval width such that at the maximum of one Gaussian the adjacent Gaussians are at 13% of their maximum values. This is also the default behaviour of *AIMLESS* in the case where averaging uses the three nearest functions. This choice appears to work well in practice and we have found no reason to use a different default.

### 9.1. Results   

To demonstrate the use of a scan-varying crystal parameterization, we chose a well diffracting thaumatin crystal, from which we collected a continuous 720° data set on beamline I03 at Diamond Light Source with attenuators set to 3% transmission, delivering approximately 2.5 × 10^8^ photons s^−1^. The beam profile was measured during routine calibration to be approximately Gaussian with a cross-section at the FWHM with major and minor diameters of 80 and 20 µm, but this shape was further defined by a circular aperture with a diameter of 50 µm during data collection, limiting the horizontal size. The low photon flux was chosen to reduce the effects of radiation damage on the crystal lattice. The data were collected on a Pilatus 6M detector with shutterless operation at 40 Hz and an image rotation width of 0.1°. The crystal was considerably larger than the beam, so in order to minimize the effect of changing the illuminated volume during the course of the data collection we aligned the beam with a sharp corner of the crystal that protruded from the mounting loop. This was performed to ensure that observed changes to the unit cell are likely to be real lattice changes rather than an effect caused by sampling different illuminated volumes.

We wished to compare the results of the smooth scan-varying global refinement using *DIALS* with the usual approach of running multiple refinement tasks, in which each determines a static crystal model appropriate only within a small local block. For this purpose, the data were processed using *XDS* through *xia*2 (Winter, 2010 ▸). We also identified strong spots using *dials.find_spots* and indexed the spot centroid positions with *dials.index*, which performed global scan-static refinement of the diffraction geometry and crystal model, resulting in the identification of a tetragonal cell with parameters *a* ≃ 57.7 Å and *c* ≃ 150.0 Å. This was followed by scan-varying refinement using all indexed reflections that passed the inclusion criteria. In this case 9300 reflections close to the rotation axis were removed, followed by the rejection of 4135 reflections with the worst residuals, leaving 306 968 reflections in the working set. Each of the crystal parameters (three orientation angles and two metric parameters) was modelled using subparameters over the φ scan. A total of 117 parameters were refined using the Levenberg–Marquardt algorithm, including six detector parameters and one beam-orientation angle. The final r.m.s.d.s at convergence (after nine minimization steps) were 0.22 pixels in *X*, 0.25 pixels in *Y* and 0.14 images in φ.

**Figure 5:**
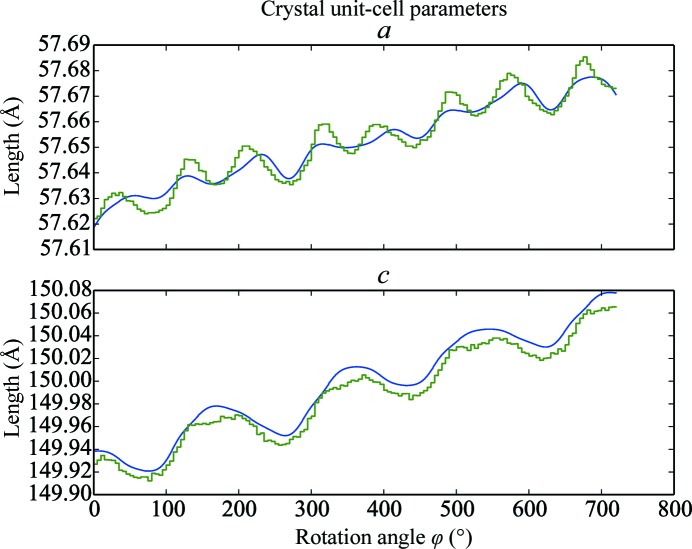
For refinement of a tetragonal thaumatin data set, scan-varying unit-cell parameters produced by the Gaussian smoothing model in *DIALS* are shown in blue, with their values refined within blocks of 5° by *XDS* shown in green. The globally determined smooth curves from *DIALS* closely match the trends observed in the independent locally determined parameter values from *XDS*.

The smoothed scan-varying unit-cell parameters are shown in Fig. 5 ▸ alongside the unit-cell parameters determined in 5° blocks by *XDS*. Comparison of the curves clearly shows that both approaches produce a model for the cell that varies with the same pattern of steadily increasing unit-cell lengths modulated by an oscillatory pattern. The overall increase in the unit-cell parameters over the course of the scan is small, with the *a* parameter increasing by about 0.05 Å, whilst the *c* cell dimension increases by a similar proportion, by approximately 0.14 Å. The period of oscillation for the *a* parameter is half that of the *c* parameter, namely 90° compared with 180°, reflecting the tetragonal symmetry of the lattice. For the higher frequency variation of *a* the smooth curve from *DIALS* has a smaller amplitude than the curve traced between the results of independent local refinement tasks from *XDS*. This is explained by the influence of each of the 22 subparameters for *a* extending well beyond the 5° block size of an individual *XDS* refinement, with this influence diminishing according to the Gaussian smoother. Compared with the set of independent refinement tasks, the smoothing of unit-cell parameters provides a constraint that includes prior knowledge in the minimization problem. This knowledge is the expectation that changes in the real lattice will occur gradually rather than abruptly. If it is suspected from an analysis of refinement residuals that the degree of smoothing is too great to capture real changes that should be modelled, then decreasing the interval width between the central positions of subparameters will enable the modelling of higher frequency variations by using more subparameters. The amplitudes of the oscillations in *c* for the *DIALS* and *XDS* curves are similar; however, the *DIALS* curve is systematically larger than that from *XDS*. The discrepancy is small, with a median value of less than 0.01 Å between the curves and a maximum of less than 0.02 Å. This difference can partly be explained by the differences in refined detector models and parallax correction between *XDS* and *DIALS*. The angle between the *c* axis and the goniometer rotation axis is about 13°, leading to higher correlations between this cell dimension and the detector parameters than for the *a* parameter. This demonstrates a difficulty in accurately measuring unit-cell parameters by fitting a model by positional or centroid refinement, as discussed in §[Sec sec7]7. Nevertheless, the discrepancy between the cells refined by *XDS* and *DIALS* should not be thought to imply a worse predictive power of one method or the other. That the *DIALS* results have a good predictive power is indicated by the low r.m.s.d.s, as reported above.

The discontinuities in the unit-cell parameter curves resulting from independent refinement tasks in blocks by *XDS* are too small in this case to have any substantial deleterious effect on the quality of integration results. However, in cases where the unit-cell parameters do change significantly during the course of data collection, for example owing to radiation damage, the step sizes of the discontinuities also increase. This may be compounded by the common practice at synchrotron facilities of making use of both coarse-grained and fine-grained parallelism, where the integration of a large sweep is split into a number of evenly sized blocks and each is integrated independently on a multi-processor computer starting from the initial model from indexing. In this situation, the size of the discontinuities may be particularly large at the borders between blocks, potentially giving rise to artefacts that would not have been observed if only fine-grained parallelism were employed. Although it is unlikely that this effect is the deciding factor affecting data quality in typical cases, it is worth noting that the approach taken by *DIALS* avoids this issue altogether by determining a smoothly varying refined model in advance of any integration. As a result, the integration program is free to take advantage of parallel execution without loss of fidelity compared with serial execution. This could be employed to provide rapid data-quality feedback utilizing massively parallel summation integration, without compromising on the quality of the diffraction-geometry model.

### 9.2. Scan-varying prediction   

Spot prediction for all reflections using a scan-varying model is more complex than for the case of a static crystal model. The smoother model requires an observed centroid value *k* along the image-number axis to produce the crystal setting matrix **A**_*k*_ for use in prediction. Clearly, observed centroids *k* exist only for those strong reflections found on the images, not for those weak reflections that were not found, so **A**_*k*_ cannot in general be produced for all reflections of interest. For some reflection **h** observed at image number *k*, the setting matrix in the laboratory frame is given by 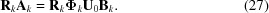



**Figure 6:**
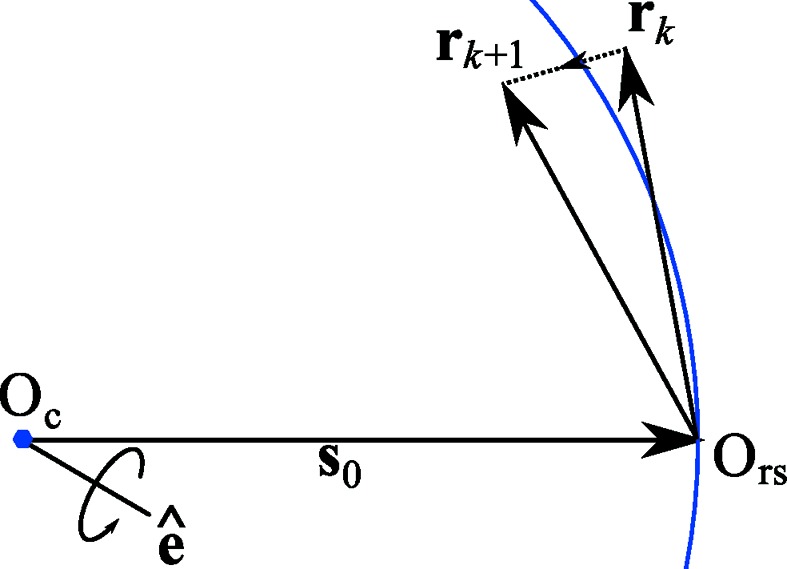
The linear transformation **T**_*k*_ operating on a reciprocal-lattice point **r**_*k*_ to produce point **r**_*k*+1_. During the course of this transformation the point crosses the Ewald sphere. The meanings of the symbols are retained from Fig. 1 ▸.

In (13), the position within the scan affects only **R**_φ_, and **r**_φ_ takes a circular path through reciprocal space from **r**_φ_ = **R**_φ_ **Ah**. In contrast, for the general scan-varying case, the vector **r**_*k*_ = **R**_*k*_ **A**_*k*_ **h** will follow a path that deviates from a circle by changes in the effective rotation axis owing to variation in **Φ**_*k*_ and changes in the lattice metric owing to variation in **B**_*k*_. Despite this complication, the path can be sampled at any point *k*. Therefore, the path can also be approximated *via* a series of linear transformations in steps that are small enough that the linear approximation is appropriate. For example, the transformation **T**_*k*_ combines the change in the crystal orientation and lattice from one image to the next,
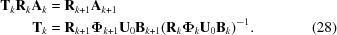
All of the terms on the right of (28) are known, thus **T**_*k*_ can be calculated. **T**_*k*_ can be applied to a reciprocal-lattice vector at image number *k* to take it to its position at image number *k* + 1, 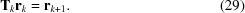
Some reciprocal-lattice points may cross the Ewald sphere during the transformation, as shown in Fig. 6 ▸. This is easy to determine: for each candidate reflection **h**, calculate **r**_*k*_, then 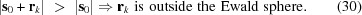
If a reflection changes status (outside to inside or *vice versa*) after application of the transformation **T**_*k*_ then it is predicted to be present on image *k*. In fact, under the linear approximation, the true path in reciprocal space has been approximated by the segment **Δr** = **r**_*k*+1_ − **r**_*k*_. The distance to the Ewald sphere from **r**_*k*_ along this segment is found by solving the following for β: 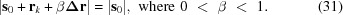
The predicted image centroid is then given by 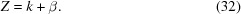



The algorithm for scan-varying reflection prediction in *DIALS* performs these steps for candidate reflections on each image of the scan. For efficiency, it is important to preselect the list of candidates to remove the great majority of reflections that are not likely to cross the Ewald sphere over the range of that single image. A version of Reeke’s algorithm for reflection-list generation (Sweet, 1986 ▸) is used to form a shell of reciprocal-lattice points close to the Ewald sphere, within a resolution-limiting sphere, for each image. Only these reflections are tested for passage across the Ewald sphere. The version of Reeke’s algorithm within *DIALS* is based on that included in *MOSFLM*, modified to take advantage of the generalized vector geometry of the *DIALS* framework.

## 10. Error estimates for refined parameters   

When any nonlinear least-squares algorithm is used to minimize the target function, the errors on the refined parameters may be estimated by the standard procedure of inverting the normal matrix. As these parameters may not necessarily be relevant outside of refinement, it is more useful to convert these to error estimates of the model states (see Table 1 ▸ for definitions). This is achieved by the usual procedure for error propagation, as detailed in Appendix *C* [App appc], and for the crystal model estimated standard deviations of the real space unit-cell parameters may be derived.

While these error estimates must be validated by experiment, it is technically difficult to obtain real data sets drawn from a true population differing only by random noise. While we could use synthetic data from a simulation procedure, such as that described by Diederichs (2009 ▸), this does not necessarily include all of the subtleties of the features observed in real experimental data. In response to this we have devised a procedure to create ‘semi-synthetic’ data sets, described as follows.

30 low-dose data sets (*i.e.* a ‘large’ number) were recorded sequentially on beamline I04 at Diamond Light Source with identical data-collection parameters (1027 images per data set from a Pilatus 6M detector with an image width of 0.15° and 1% beam transmission at a wavelength of 1.2 Å). Scaling all of the data together indicated that radiation damage across the 30 sweeps was small; however, some systematic differences between them remained owing to factors such as beam-intensity variation.

The photon counts from these 30 ‘original’ sweeps were then ‘reshuffled’ to create a population of 30 equivalent ‘new’ data sets (for convenience, to allow reuse of the image headers) by considering every active pixel in the data set (*i.e.* each of around six billion) independently using the following procedure. Firstly, create a summed data set, which we call ‘total’:
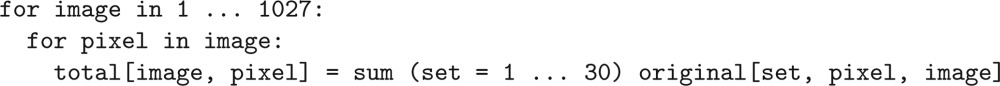


Then create 30 ‘new’ data sets with every pixel set initially to 0, and randomly redistribute each photon count from every pixel of the ‘total’ set to the same pixel position in one of the 30 ‘new’ sets:
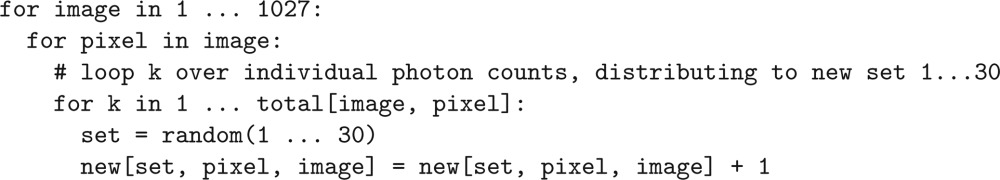


This process therefore divides individual photon measurement events in the ‘total’ data set into 30 equivalent data sets, each with approximately 1/30th of the counts, that differ only by random shot noise and can be considered statistical replicates truly drawn from a parent population. This procedure is valid, as for photon-counting detectors each photon-measurement process is independent. Systematic experimental effects such as parallax shifts, detector sensitivity and sample absorption remain, ensuring that these data sets give relevant insight into the analysis process.

Spots were found in all of the reshuffled data sets using the program *dials.find_spots*. The mean highest resolution of these spots accepted for use in refinement across all data sets was 1.7 Å, with 95% of all spots used in refinement within 2.3 Å. The first of these data sets was indexed by *dials.index*, which identified a tetragonal lattice. We took this solution as a starting point to index all 30 data sets, but with the lattice symmetry now relaxed to produce a triclinic solution so that we could investigate the errors on the unit-cell angles. This procedure ensured that the same basis was used for each data set. Global scan-static refinement was performed for each data set using all available reflections and the default outlier-rejection algorithm (the MCD method at the 97.5% quantile threshold). Each refinement used a similar number of reflections and resulted in comparable final r.m.s.d.s, indicating a high degree of similarity between the models at convergence. For comparison, we then repeated the procedure from the point of indexing onwards but this time retaining the tetragonal symmetry. The results are summarized in Table 2 ▸. For each unit-cell parameter, the observed scatter of the refined values across all 30 runs is comparable to the mean of the error estimates from each run. It is therefore clear that the mean error estimates are a reasonably good predictor of the observed scatter across the replicate data sets. Indeed, for all parameters the standard deviation of the 30 error estimates (not shown) was less than 0.3% of the value of the error estimates, so any one of the estimates from refinement of a single data set is seen to be a fair estimate of the observed scatter across data sets that differ in random noise.

Despite the predictive power of the error estimates reported after refinement, these must be treated with care and understood for what they are, which are predictions of the variability of the model in the presence of purely random errors and not a measure of the absolute correctness of that model. Here, it can be seen that the error estimates for each of the unit-cell angles in the triclinic runs are more than an order of magnitude smaller than the discrepancy between the refined values of these angles and the expected value of 90° considering the known tetragonal lattice. In this case, overparameterization of the crystal allowed the refinement to fit a model with a high reported precision, but inaccurate on the scale of that precision. Despite this overfitting, the r.m.s.d.s from the triclinic refinements are similar to those from the tetragonal refinements, indicating that either case provides a good model to predict the location of spots throughout the scan. For the tetragonal case, the error estimates on the *a* and *b* parameters are correctly equal, and the error estimate of the angle parameters is correctly zero. At no point during the error-propagation procedure presented in Appendix *C* [App appc] are the symmetry constraints explicitly enforced, but these are carried through implicitly by use of the correct derivatives.

## 11. Conclusions   

A comprehensive description of diffraction-geometry refinement within the *DIALS* framework has been given and the tools made available within the command-line program *dials.refine*. This program builds upon a simple but general model for the prediction of reflection central impacts and minimization of a least-squares residual by providing flexible parameterization, choices of minimization algorithm and outlier-rejection method, global scan-varying refinement of the crystal and joint refinement of multiple experiments. Examples of these advanced features have been shown, as well as an analysis of the propagation of errors using a novel method for drawing statistical replicate data sets from real experimental data. The design of the software is explicitly intended to facilitate extensibility and modification, and to provide a solid platform from which future research into advanced methods of diffraction-geometry modelling may be performed. 

## Figures and Tables

**Table 1:** Default parameterization in *DIALS* for scan-static refinement using a single-panel detector The vector 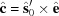 is defined here for convenience.

Parameterization	Model state	Parameters	Action
Beam	**s**_0_	μ_1_	Rotation about 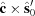
		μ_2_	Rotation about 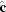
		ν	Set length of *s*_0_ (wavenumber)
Crystal orientation	**U**	φ_1_	Rotation about laboratory 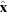
		φ_2_	Rotation about laboratory 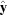
		φ_3_	Rotation about laboratory 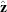
Crystal unit cell	**B**	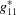	Set metrical matrix elements
		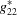	
		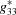	
		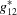	
		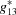	
		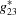	
Detector	**d**	*p*_0_	Set distance along 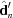
		*t*_1_	Translation along 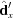
		*t*_2_	Translation along 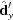
		τ_1_	Rotation about 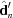
		τ_2_	Rotation about 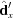
		τ_3_	Rotation about 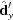

**Table:** The mean value of each of the refined unit-cell parameters is shown along with its standard deviation, which can be compared with the mean of the estimated standard deviations reported for each refinement run.

	Triclinic		Tetragonal	
	Minimum	Maximum	Minimum	Maximum
No. of reflections	83632	84265	83911	84484
R.m.s.d. in *X* (pixels)	0.231	0.233	0.235	0.238
R.m.s.d. in *Y* (pixels)	0.195	0.197	0.201	0.203
R.m.s.d. in φ (images)	0.147	0.149	0.150	0.152

**Table:** 

	Triclinic			Tetragonal		
	Mean value	Sample s.d.	Mean e.s.d.	Mean value	Sample s.d.	Mean e.s.d.
*a* (Å)	121.44796	0.00069	0.00081	121.48944	0.00091	0.00062
*b* (Å)	121.46668	0.00068	0.00065	121.48944	0.00091	0.00062
*c* (Å)	56.99998	0.00036	0.00040	57.01478	0.00048	0.00036
α (°)	89.98317	0.00020	0.00022	90	0	0
β (°)	90.00865	0.00020	0.00020	90	0	0
γ (°)	89.99427	0.00015	0.00020	90	0	0

## Appendix A First derivatives of the prediction formula

### A1. Derivatives of the rotation angle

Whereas the vector **v** from (5) provides a way to express the *X* and *Y* coordinates as a function of a parameterized model, there is no equivalent expression for the rotation angle φ immediately available. Following Bricogne (1987 ▸), such an expression is obtained using the implicit function theorem, which states that if there is a continuous function *G*(φ, *p*) = *c*, where *c* is a constant, then the function φ(*p*) has derivatives with respect to some abstract parameter *p* given by (∂/∂*p*)φ(*p*) = −(∂*G*/∂*p*)/(∂*G*/∂φ).

Here, the continuous function *G* is the diffraction condition, which is equivalent to

Changes to a parameter *p* imply changes to the rotation angle φ to keep the reciprocal-lattice point in the diffracting position. Thus,

As neither the interplanar spacing nor the direction of the beam vector are affected by rotation of the crystal, then (∂/∂φ)(**r**_φ_ · **r**_φ_) = 0 and ∂**s**_0_/∂φ = 0. Now consider ∂**r**_φ_/∂φ. Referring to (9), this can be shown to be as follows,

but noting that the triple product

it is seen that

Putting this into (34) and simplifying gives

From (8), at any fixed φ

therefore

This equation factors the calculation of derivatives of the rotation angle into independent parameterizations. The term ∂**s**_0_/∂*p* refers to the beam parameterization, while ∂**r**_0_/∂*p* refers to the crystal parameterization. The latter can be expanded further as

where **U**_0_ is the datum crystal orientation matrix determined at the moment when the crystal orientation parameters are defined and **ΦU**_0_ is the crystal orientation including all ‘misset’ angles determined after some steps of refinement have occurred. Hence,

where ∂**Φ**/∂*p* refers to the crystal orientation parameterization and ∂**B**/∂*p* refers to the crystal unit-cell parameterization.

### A2. Derivatives of positions in detector space

From (7), the partial derivatives with respect to an abstract parameter *p* are constructed by the quotient rule,

From (5), the partial derivatives ∂*u*/∂*p*, ∂*v*/∂*p* and ∂*w*/∂*p* are elements of

where the standard formula for the derivative of an inverse matrix was used, ∂**M** ^−1^/∂*t* = −**M** ^−1^(∂**M**/∂*t*)**M** ^−1^, but as **v **= **Ds**_1_ and **s**_1_ = **r**_φ(*p*)_ **s**_0_ this can also be written as

Here, we are careful in the notation to make explicit the fact that the rotation angle φ is a function of the parameters *p* necessary to satisfy the Bragg condition. In §[Sec seca1]A1 the partial derivatives at a particular value of φ were being evaluated. In contrast, here the partial derivatives at the central impact (*X*, *Y*) are required and it cannot be assumed that φ is fixed. Rather, the impact (*X*, *Y*) has an indirect dependence on φ caused by the diffraction condition, which constrains **r**_φ(*p*)_ to lie on the Ewald sphere. (39) does not apply here. Instead, we proceed by considering **r**_φ(*p*)_ as a composition of functions. Starting with (8), it is seen that **r**_0_ is a function of the crystal parameters and that **R**_φ_ is a function of the rotation angle φ, which is itself a function of beam and crystal parameters. In this situation a multivariable chain rule is used to differentiate **r**_φ(*p*)_ with respect to some variable *p*,

For those parameters *p* for which the gradient ∂φ/∂*p* (calculated in equation 40) is nonzero (*i.e.* beam and crystal parameters), this expression modifies (39) to encode the constraint that the only changes to **r**_φ(*p*)_ allowed by the reflection-prediction equation are those that leave the vector on the surface of the Ewald sphere. Incorporating (46) into (45) gives

This equation decomposes the calculation of derivatives into separate, independent parameterizations of the model. The ∂**d**/∂*p* term refers to the detector parameterization, while the ∂**r**_0_/∂*p* and ∂**s**_0_/∂*p* terms refer to the crystal and beam parameterizations, respectively. As before, the crystal parameterization is expanded further by (42).

## Appendix B First derivatives of model states

### b1. Beam parameterization

The composition of a new beam model state from its parameters is given by (11). The first derivatives of the state with respect to its parameters are

Calculation of  and  may proceed using the matrix form of Rodrigues’ rotation formula,

where **E** is the skew-symmetric cross-product matrix,

and *e_i_* are elements of , the axis of rotation,

### b2. Crystal orientation parameterization

The crystal orientation matrix is separated into the product of a variable misset matrix (see equation 12) and a fixed datum orientation,

The first derivatives are

Expansion of the rotation-matrix derivatives follows (53).

### b3. Crystal unit-cell parameterization

The crystal unit-cell parameterization relies on code already in *cctbx* under the *rstbx* package to calculate the derivatives of the model state **B** with respect to the free elements  of the reciprocal metric matrix, **G***, where

### b4. Detector parameterization

The detector state matrix **d** is split into vectors ,  and **d**_0_ for the purposes of calculating derivatives (see equation 2). The detector-frame basis vectors are not affected by the translational parameters, therefore

Starting from (14), derivatives of the in-plane detector basis vectors with respect to the orientational parameters are calculated

Further expansion follows (53). Using these results, derivatives for the detector normal direction are calculated, thus completing derivatives of the basis set

Composition of the detector origin from new parameter values concludes with (23). The derivatives of this with respect to the translational parameters are

The derivatives with respect to orientational parameters are

Combining (59), (60), (62)–(67) [Disp-formula fd63] [Disp-formula fd64] [Disp-formula fd65] [Disp-formula fd66] [Disp-formula fd67] and (71)–(76) [Disp-formula fd72] [Disp-formula fd73] [Disp-formula fd74] [Disp-formula fd75] [Disp-formula fd76] gives the derivatives of **d** with respect to its parameters.

## Appendix C Error propagation

Nonlinear least-squares minimization algorithms provide an estimated covariance matrix between all parameters, **V**_*p*_, as the inverted normal matrix from the final step of refinement. We are interested in converting this into errors in terms of the elements of the vector or matrix models for the beam, crystal and detector. We start by selecting only the rows and columns of **V**_*p*_ corresponding to parameters of the particular model of interest. For illustration here the model for the crystal unit cell is used with state **B**, but the working is equivalent for any of the models. The covariance matrix of parameters of the unit-cell model is here denoted  and we wish to propagate errors to the elements of **B**. The standard first-order approximation is used to do so (Arras, 1998 ▸), but the model state **B**, which is a matrix rather than a vector, is first ‘flattened’ to choose the order of rows and columns in . Any ordering of elements could be used to do this, but we use row-major order. The covariance matrix of elements of **B** is then given by

where  is the Jacobian with elements ∂*B_i_*/∂*p_j_*, which are elements of the derivatives of the model state with respect to its parameterization. The variances of elements of **B** can then be obtained by taking the diagonal of **V**_*B*_ and reversing the row-major order flattening operation.

This procedure is sufficient for reporting errors in models in terms of their model state elements rather than the particular parameterizations that were chosen in refinement. However, for the example we have chosen, unit-cell parameterization, we prefer to propagate errors further so that we can report standard deviations in terms of the real-space unit-cell parameters.

### c1. Unit-cell parameters

(77) provides **V**_*B*_, the covariances between elements of **B**. Recognizing that **B** consists of the reciprocal-lattice vectors **a***, **b*** and **c***, quantities related to the reciprocal lattice can easily be derived, such as the variances of the reciprocal unit-cell dimensions, ,  and . However, it is more convenient for the user if estimated errors are expressed in terms of the real-space cell. This requires further steps of error propagation, starting with the conversion between **B**, the reciprocal-space orthogonalization matrix, and **O**, the real-space orthogonalization matrix:

Propagation of errors must therefore go through a transpose and a matrix inverse operation. The first of these is merely a permutation of elements,

where, for the choice of flattening by row-major ordering,

Accounting for the matrix inverse operation is more involved. For this, the method of Lefebvre *et al.* (2000 ▸) is used. The elements of **V**_*O*_, the covariance matrix of elements of **O** written out in row-major order, are calculated according to this formula, where element indices are shown inside square brackets,

and index values are chosen from α, β, *a*, *b* ∈ {1, 2, 3}. Having obtained **V**_*O*_, the final steps of error propagation are performed to derive variances of each of the real-space unit-cell parameters. For each parameter consider the function that converts the elements of **O** into the value of that parameter. For the unit-cell length *a* this function is simply

The Jacobian of this function is a 1 × 9 matrix,

which is used to calculate the variance in *a* by

The calculations for the other lengths, *b* and *c*, follow by analogy, with

For the unit-cell angle α the following function is used:

The derivatives of α with respect to elements *a_i_*, *b_i_* and *c_i_* are given by

These formulae are used to construct **J**_α_ and hence calculate σ_α_^2^ from

The calculations for σ_β_^2^ and σ_γ_^2^ follow equivalently.

### c2. Simulation results

The procedure presented in §[Sec secc1]C1 to propagate errors from **V**_*B*_ to errors in the real-space unit-cell parameters can easily be tested against simulation. As an example, we took **B** and **V**_*B*_ after refinement for one of the 30 data sets presented in §[Sec sec10]10 for the case where the lattice was treated as triclinic. We then used the *rmnorm* function from the *lmf* package (Engen *et al.*, 2012 ▸) for the R language to generate one million matrices such that the matrix elements were normally distributed around the elements of **B** with covariances described by **V**_*B*_. For each trial **B**_*i*_ the real-space unit-cell parameters were calculated according to (78), (82) and (87) *etc*. We then compared the sample standard deviation of the one million sets of unit-cell parameters with the estimated standard deviation resulting from the error-propagation procedure. For each parameter the difference between the estimated standard deviation from error propagation and the observed sample standard deviation from simulation was within 0.1%, thus demonstrating that the procedure works as expected when the data are normally distributed.
